# Geographic, Racial/Ethnic, and Sociodemographic Disparities in Parent-Reported Receipt of Family-Centered Care among US Children

**DOI:** 10.1155/2015/168521

**Published:** 2015-12-17

**Authors:** Romuladus E. Azuine, Gopal K. Singh, Reem M. Ghandour, Michael D. Kogan

**Affiliations:** Office of Epidemiology and Research, Maternal and Child Health Bureau, Health Resources and Services Administration, US Department of Health and Human Services, 5600 Fishers Lane, Room 10-77, Rockville, MD 20857, USA

## Abstract

This study examined geographic, racial/ethnic, and sociodemographic disparities in parental reporting of receipt of family-centered care (FCC) and its components among US children aged 0–17 years. We used the 2011-2012 National Survey of Children's Health to estimate the prevalence and odds of not receiving FCC by covariates. Based on parent report, 33.4% of US children did not receive FCC. Children in Arizona, Mississippi, Nevada, California, New Jersey, Virginia, Florida, and New York had at least 1.51 times higher adjusted odds of not receiving FCC than children in Vermont. Non-Hispanic Black and Hispanic children had 2.11 and 1.58 times higher odds, respectively, of not receiving FCC than non-Hispanic White children. Children from non-English-speaking households had 2.23 and 2.35 times higher adjusted odds of not receiving FCC overall and their doctors not spending enough time in their care than children from English-speaking households, respectively. Children from low-education and low-income households had a higher likelihood of not receiving FCC. The clustering of children who did not receive FCC and its components in several Southern and Western US states, as well as children from poor, uninsured, and publicly insured and of minority background, is a cause for concern in the face of federal policies to reduce health care disparities.

## 1. Introduction

Family-centered care (FCC) is a collaborative approach to health care where family perspective is central to health care decision-making and represents a core aspect of a system of health care that focuses on the needs of all children in the United States [1]. Health care delivery that is family-centered can improve both the quality of health care services received by children and their families and patient/family satisfaction with those services [1, 2]. FCC is also an important aspect of the quality of a medical home which is defined as a model of primary care that is accessible, continuous, comprehensive, family-centered, coordinated, compassionate, and culturally effective [3].

For nearly five decades since it was first espoused, FCC, as a component of the medical home, has been advanced as an important part of quality care delivery by leading health professional associations, family advocates, and public health policy organizations, including the American Academy of Pediatrics, American Academy of Family Physicians, American College of Physicians, and the American Osteopathic Association, the Institute of Medicine, and the federal Maternal and Child Health Bureau (MCHB) at the Health Resources and Services Administration (HRSA) [4–6]. In fact, Healthy People 2020 identifies the ability to find a health care provider with whom the patient can communicate and trust as one of the public policy linchpins for improving access to comprehensive, quality health care services [7]. FCC has been shown to reduce cost for individuals, families, employers, and government and support evidence-based interventions that address multifaceted aspects of socioeconomic and neighborhood determinants of health [8, 9]. The Institute of Medicine identifies family-patient-centered care as one of the six characteristics of quality care that the US health care system should strive to deliver to all patients [6]. In addition, family-patient-centered care is an essential feature of primary care whose mission is to provide entry into the health service system, provide person-focused care over time, provide care for all but very uncommon or unusual conditions, and coordinate or integrate care provided elsewhere or by others [10].

Although FCC has been acknowledged by health care professionals and policy makers as a marker of quality health care delivery and integral to improved health care delivery, in-depth population-based and national studies examining the different components of FCC are lacking. Existing studies have explored FCC among select groups of children, including children with specific medical/health conditions, children with special health care needs, children from predominantly English-speaking families, children from high income families, or those from specific regions of the country [8, 11–13]. Little is known about geographic, racial/ethnic, and socioeconomic disparities in the receipt of FCC among all children in the United States (US). Furthermore, while public policy discussions on integrating family opinions and perspectives into the health care of children in the US have focused on increasing the number of children with access to a medical home and selected components, such as care coordination, less attention has been paid to the experience of families with health care providers within the context of the medical home [14]. Given the benefits of FCC for children and their families, an understanding of the determinants of access to FCC for parents and children at the national-level is important in addressing disparities in family experiences within the medical home. In addition, understanding geographic and sociodemographic disparities in access to FCC can provide pertinent information to program planners and policy makers in the design and targeting of interventions and services to serve children and families [15]. The purposes of this study were twofold: (1) to estimate the prevalence of parent/family-reported receipt of FCC among children in various socioeconomic and demographic groups and across the 50 states in United States (US) and the District of Columbia (DC) and (2) to identify sociodemographic groups and states with lower receipt of FCC among US children.

## 2. Methods

### 2.1. Data Source

Data for this study were from the 2011-2012 National Survey of Children's Health (NSCH). With funding and direction from HRSA/MCHB, the NSCH is a nationally representative survey designed to assess the physical and emotional health of 95,677 children aged 0–17 years, as well as factors that may relate to child well-being, including medical homes, family interactions, parental health, school and after-school experiences, and neighborhood characteristics [16]. The 2011-2012 NSCH is a cross-sectional telephone survey of US households with at least one resident child aged under 18 at the time of the interview. The survey used a list-assisted random-digit-dial (RDD) sample of landline telephone numbers, supplemented with an independent RDD sample of cell-phone numbers. Using a complex survey design, with stratification by state and sample type (landline or cell-phone), telephone numbers were called and screened for residential status and the presence in the household of children who were aged 0–17 years at the time of the call. One child was randomly selected to be the subject of the detailed interview if more than one child lived in the household. In households with one child, that child was selected to be the subject of the detailed interview. One parent or guardian with knowledge of the health and health care of the sampled child in the household completed the survey. The survey was conducted in all the 50 states and DC between February 2011 and June 2012 using the State and Local Area Integrated Telephone Survey mechanism [16]. All survey data were based on parental reports. Interviews were conducted in English, Spanish, and four Asian languages. The interview completion rate for the 2011-2012 NSCH, a measure of the response rate indicating the percentage of completed interviews among known households with a child under 18, was 54.1% for the landline sample and 41.2% for the cell-phone sample [16]. Detailed information about the NSCH can be found elsewhere [16]. The NCHS Research Ethics Review Board approved all data collection procedures for the survey.

### 2.2. Study Variables

The dependent variable in our study was the receipt of FCC and its five components. Children receiving FCC and its components were defined as children whose parents/guardians responded positively to the survey questions inquiring on whether their child's doctor or health care provider usually or always (1) listened carefully; (2) were sensitive to the family's values and customs; (3) made the family feel like a partner in the child's care; (4) provided needed information; and (5) spent enough time with the family. One of our primary independent variables was state of residence. Other key independent variables included race/ethnicity, household language use, and household education and income levels. In all, the following socioeconomic, demographic, and health services variables were considered as covariates: child's age (0–5, 6–11, 12–17 years); child's sex; race/ethnicity (Hispanic, Non-Hispanic White, Non-Hispanic Black, mixed race, and other); household composition (two-parent biological household, two-parent stepfamily, single mother, or other); child health status (child with special health care needs or nonspecial health care needs); state of residence (50 US States and DC); place of residence (metropolitan or nonmetropolitan); primary language spoken at home (English or any other language); highest parental education in years of school completed (<12, 12, 13–15, and 16+); household poverty status measured as a ratio of family income to the federal poverty level (FPL) (<100%, 100–199%, 200–399%, and ≥400%) [17]; and type of health insurance (private, public, both public and private, and uninsured).

### 2.3. Statistical Analysis

We modeled the odds of not receiving FCC and its components for 91,001 children as a function of the geographic and sociodemographic covariates described above. Children with missing data on FCC were excluded from all analyses [16]. Prevalence (%) estimates of FCC were computed for all covariates and for children in all 50 states and DC. The *χ* ^2^ statistic was used to test the overall association between each covariate and FCC receipt. Multivariate logistic regression was used to examine the adjusted association between covariates and the binary outcome variables of overall FCC receipt and its five components. To account for the complex sample design of the survey, SUDAAN software [18] was used to conduct multivariate logistic analyses and to determine prevalence estimates and their standard errors.

## 3. Results

**Figure 1:**
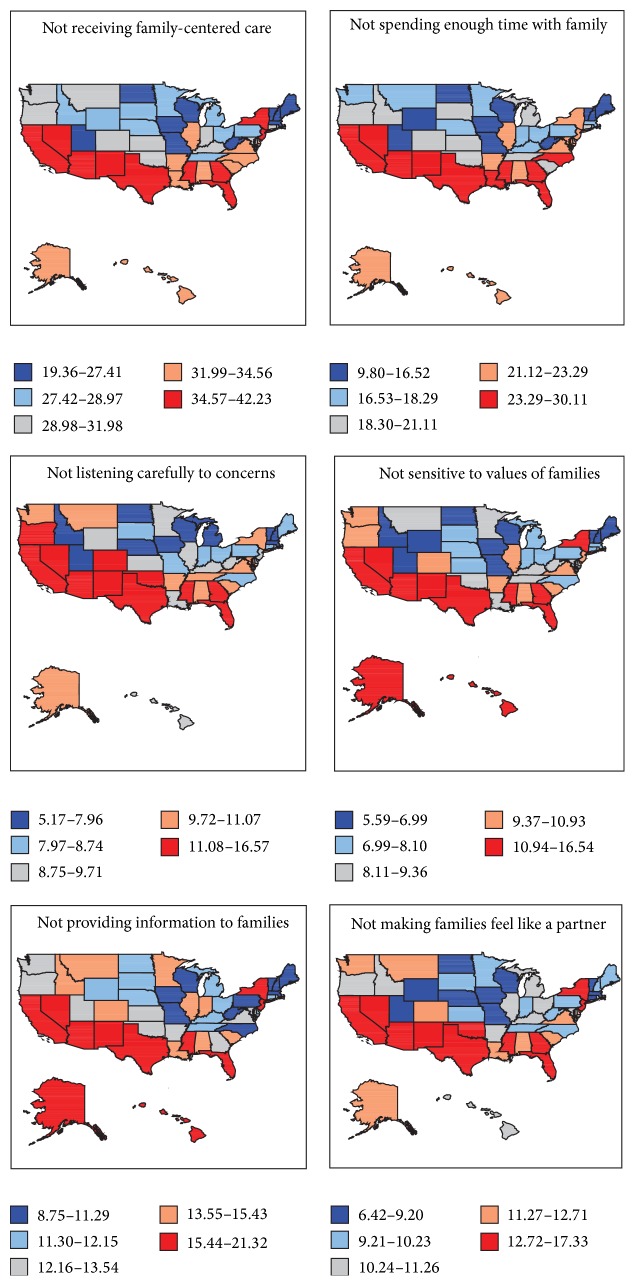
Quintile maps showing state variation in the prevalence of not receiving family-centered care, United States: the 2011-2012 National Survey of Children's Health.

Table 1 presents the observed prevalence of not receiving FCC and its five components, according to parental report. Nationally, 33.4% of children did not receive FCC. In all, health care providers did not spend enough time with 22.5% of children's families; did not listen carefully to the concerns of 10.6% of families; were not sensitive to the values of 10.8% of families; did not provide information to 14.7% of families; and did not make 12.3% families feel like a partner. The maps in Figure 1 show relatively higher rates of not receiving FCC and its components in a number of Southern and Western states and lower rates in the Midwestern states. In particular, children in Arizona, California, Mississippi, New Mexico, New York, Nevada, and Texas had the lowest receipt of FCC and its components. The percentage of children who did not receive FCC ranged from a low of 19.4% in Vermont to a high of 41.0% in Arizona and 42.2% in California. The percentage of children whose health care provider did not spend enough time with them ranged from a low of 9.8% in Vermont to a high of 30% in California and 30.1% in Arizona. Parents reported that health care providers did not listen carefully to the concerns of 14.4%, 14.6%, and 16.5% of children in Mississippi, California, and Nevada, respectively; this percentage was the lowest in Vermont and Massachusetts (5.2%). The percentage of children whose families did not feel like partners in their care were the highest in Arizona (15.8%), Mississippi (15.9%), California (16.7%), and Nevada (17.3%) and the lowest in Vermont (6.9%) and Wisconsin (6.4%).

An overwhelming percentage of minority children, children from non-English-speaking households, and those from poor families did not receive FCC and its components. For example, 50% of Hispanic and 43.8% of non-Hispanic Black children did not receive FCC. Health care providers did not spend enough time with 39.1% and 31.4%, respectively, of Hispanic and non-Hispanic Black children. Approximately, 62.0% of children from non-English-speaking families and 50.6% of children from households living below 100% of the federal poverty threshold were reported not to receive FCC. Approximately, 59.0% of children whose parents had less than a high school education were reported not to receive FCC, compared with 26.3% of children whose parents had a college education.

Table 2 presents the unadjusted odds of access to FCC and its components, showing marked variations by sociodemographic and geographic variables.  Table 3 shows state variations in selected socioeconomic and demographic characteristics, which may contribute to the observed geographical disparities in the prevalence and odds of access to FCC and its components shown in Tables 1 and 2. The percentage of non-white minority children was the highest in the Southern and Western regions of the United States and the lowest in the Midwest. More than 82% of children in Hawaii were of minority ethnicity, compared with only 12% in Maine (Table 3). The Western states had the highest percentage of non-English-speaking households; the percentage varied from <1% for West Virginia and Vermont to a high of 35.4% for California. Low-income and low-education households were most prevalent in the Southern and Western United States. Approximately 33% of the households in Mississippi were below the poverty level, compared with 10% of the households in Wyoming. Approximately 19% of the households or parents in Nevada and California lacked a high school education, compared with only 2% in New Hampshire and North Dakota.

Even after controlling for socioeconomic and demographic covariates, wide disparities in the receipt of FCC and its components persisted for children in a collection of states, for racial/ethnic minority children, and for children from low-income and low-education households (Table 4). For example, children in Arizona, Mississippi, Nevada, California, New Jersey, Virginia, Florida, and New York had at least 1.51 times higher adjusted odds of not receiving FCC than children in Vermont. Families of children in New York and New Jersey had 1.43 (AOR: 1.43; CI: 1.01–2.03) and 1.64 (AOR: 1.64; CI: 1.15–2.34) times higher adjusted odds, respectively, of not feeling like partners in their child's care than families in Vermont. Children in Maryland and Mississippi had 1.52 (AOR: 1.52; CI: 1.10–2.10) and 1.91 (AOR: 1.91; CI: 1.42–2.58) times higher odds, respectively, of their health care providers not spending enough time with them during their care, while children in New York and Mississippi had at least 1.49 and 1.58 times higher odds of doctors not being sensitive to their values and customs than those in Vermont, respectively. Children from non-English-speaking households had 2.23 and 2.35 times higher adjusted odds of not receiving FCC and their health care providers not spending enough time in their care than children from English-speaking households.

Before statistical adjustments, non-Hispanic Black and Hispanic children had 2.71 and 3.48 times higher odds, respectively, of not receiving FCC than non-Hispanic White children (Table 2). However, these disparities were somewhat attenuated after adjustment for covariates. In the fully adjusted model, non-Hispanic Black and Hispanic children had 2.11 and 1.58 times higher odds of not receiving FCC than non-Hispanic White children (Table 4). Non-Hispanic Black and Hispanic children, respectively, had 2.48 (AOR: 2.48; CI: 2.21–2.79) and 1.91 (AOR: 1.91; CI: 1.66–2.19) times higher adjusted odds of their parents reporting that health care providers did not spend enough time with them during their care than non-Hispanic White children. Compared to non-Hispanic White children, non-Hispanic Black and Hispanic children had 2.11 (AOR: 2.11; CI: 1.79–2.49) and 1.69 (AOR: 1.69; CI: 1.39–2.07) times higher odds of their parents reporting that health care providers were not being sensitive to their family's customs and values, respectively. Children from families living below 100% of the federal poverty line had 2.08 times or higher odds of not receiving all but one of the five components of FCC compared to their most-affluent counterparts. The only exception was the odds of their health care providers not providing needed information, which was 1.98 (AOR: 1.98; CI: 1.66–2.37) times higher for poor children than for children from the most-affluent families. Children without health insurance had 2.83 times higher odds of not receiving FCC than those with private insurance.

## 4. Discussion 

Our study is, to our knowledge, probably among the first to document the extent of state-level disparities in receipt of FCC and its components among US children and to determine the degree to which individual-level socioeconomic and demographic factors explain the observed geographic disparities using a nationally representative dataset. Although our study is focused on children and families in the United States, our findings have far-reaching national and international implications. Globally, family and patient-centered care is a critical component of a high functioning primary care which in turn has the potential to reduce the adverse health effects of social inequalities associated with income distribution and resource distribution [10]. The World Health Organization continues to advance the reorganizing of health services such as primary care around the needs and expectations of people, thus making them more socially relevant and responsive to the changing world while producing better health outcomes as one of the four sets of primary care reforms around the world [19]. Over the last three decades, nearly every wealthy country in the Organisation for Economic Cooperation and Development (OECD) has implemented reforms that aimed, among other things, at achieving high-quality primary care that drives improved population health outcomes [20]. In European countries such as Netherlands, experts have called on primary care to respond appropriately, both in expertise and supply, to the increasing ethnic and cultural diversity of European countries [21]. This clarion call against the background of our findings that parents of children from non-English-speaking households and from ethnic minority backgrounds such as non-Hispanic Black and Hispanic children reported disproportionately higher odds of parents reporting that health care providers were not being sensitive to their family's customs and values is poignant. In poor non-OECD countries, such as Africa, equitable and sustainable access to properly functioning health systems for decades has eluded scores of economically deprived populations and those living in rural areas [22]. A health system that optimally delivers in the components of family-centered care can help address the widening disparities in access and prohibitive costs of basic health care which culminates in the deterioration of people's social status and crunching poverty [22].

Given the importance of FCC, both as a quality indicator and a measure of the family's relationship with their child's health care provider, our findings reveal troubling patterns of disparities in the experience of minority and poor children with health care providers. We found that parents of as many as 12.3% of children nationally and from 15.1% to 17.3% in Florida, Arizona, Mississippi, California, and Nevada did not feel like a partner in their child's care. A recent study reported that 16% of parents reported that they did not partner with their health care providers in making decisions on the desired outcome for their child [11]. These estimates are fairly similar even though our study is national in scope covering all children, whereas the latter study presents data for a special population of children with specific health conditions and from predominantly middle-class families [11]. We found that health care providers were not sensitive to the customs and values of as many as 15.1% and 16.5% of children in Mississippi and Nevada, respectively. These estimates are significantly lower than those reported in a recent study among children with neurodevelopmental disabilities where as many as 95% of families reported that their child's health care provider did not consider their cultural preferences when linking them to services that may be needed by their children [11]. It is conceivable that health care providers are not ethnically and culturally as diverse as the families they are serving who are increasingly becoming diverse by the day. These estimates are troubling given that minorities are projected to constitute almost half of the US population by 2050 [23]. Demographic changes and their attendant diversities in customs, languages, and cultural values present complex challenges that health care providers will face more and more.

The clustering of nonreceipt of FCC and its components within the same states of the Southern and Western United States such as Arizona, California, Mississippi, and Nevada with an increasing number of minority children is a cause for concern in the face of federal policies to reduce health care disparities. A prior study has reported a similar pattern of disparities in access to medical home and its components [8]. More troubling is that the national estimates for the overall receipt of FCC were largely unchanged in the last five years when the last NSCH was conducted [24]. Persistent disparities among ethnic minority children in various states—big and small alike—indicate that inequalities in receipt of FCC are likely the result of demographic and socioeconomic differences among children rather than geography [15]. A majority of these states such as Arizona, Nevada, and California have a large Hispanic population which might present language barriers thus culminating in the estimates in our analysis. However, this argument pales given that states with predominantly non-Hispanic populations such as Mississippi, Alabama, Louisiana, Maryland, and the District of Columbia also have estimates of nonreceipt of FCC greater than 32%. It is also important to note that these States have high population of African Americans. States with poor FCC outcomes for children could participate in peer-learning and quality improvement programs such as the National Improvement Partnership Network where they can learn strategies for addressing the disparities documented in our study [25].

When fully implemented within a high-quality medical home, FCC may lead to improved health outcomes, health care delivery, and health system transformation, at least in a specialized population [11]. Our findings indicate that almost half of racial/ethnic minority children and their families are not receiving the benefits of FCC. This may impede access to other needed services which has potential consequences for population health and costs to the health care system [26]. Household language is a proxy for immigrant household status and the length of stay in the US [27]. Thus, our finding that 62% of children from non-English-speaking households did not receive FCC indicates the magnitude of challenges faced by immigrants in accessing and navigating the US health care system. It could also be indicative of limited competencies of health care providers in dealing with a patient population who might have different languages, cultural needs, and preferences [28]. In the US, about 21% of 291.5 million people aged 5 years and older speak a language other than English at home [29]. Medical and allied health professional training programs should introduce the teaching of patient-communication skills and cultural competence modules in their training programs so that health care practitioners are better prepared to deal with an increasingly diverse multilingual and multicultural population in the country. Such training programs should include identification and proper use of robust FCC assessment tools such as one developed by Family Voices [30]. Health care professional organizations should work with patient organizations in making these tools available for other health care providers and supporting the use of these tools through policy statements and clinical guidelines. Further research is needed to elucidate the reasons why minority children and children from poor socioeconomic backgrounds continue to receive care that is not considered family-centered. 

*Limitations*. Our study has limitations. Children's receipt of FCC and its components is based on parental reports and may not accurately reflect the actual prevalence. Secondly, NSCH is a cross-sectional survey and, given its cross-sectional nature, we are unable to draw causal inferences from the data. Furthermore, NSCH is a household-based survey with respondents drawn from households with telephone access. It is possible that some children in transitory homes, migrants, or institutionalized children may have been excluded in the survey. The NSCH questionnaire is translated from English to Spanish and four Asian languages—Mandarin, Cantonese, Vietnamese, and Korean. The restriction to only these languages means that potential participants who speak non-Asian and non-Spanish languages may have been excluded from the survey. The survey contains subjective, parental perception, and reporting of receipt of FCC that may not be an objective measure of FCC. There is also potential recall bias for respondents answering survey questions several months after the event of interest; in this case, FCC or any of its components had occurred.

## 5. Conclusion

In conclusion, the evidence presented here suggests that the nation's aspiration for a continuously learning health care system that delivers patient-centered care, where patients' preferences are elicited, integrated, and honored, remains a work in progress [31]. It can make a difference in people's lives when health care providers listen to their patients and families, show them respect, and answer their questions in a culturally and linguistically appropriate manner [23]. The clustering of children who did not receive FCC and its components within the same states, particularly in the Southern and Western United States, as well as the disproportionate number of children from poor, uninsured, or publicly insured and of minority background, is a cause for concern in the face of federal policies to reduce health care disparities. Primary care is the cornerstone of every health care system devoted to improving health outcomes and reducing health disparities [32]. Almost one-quarter (23.6%) of children in US have at least one of a list of 18 chronic conditions which places tremendous demands on their families due to time and expense required for their care [4]. By discharging their care in a family-centered manner, pediatricians and other health care providers could be sources of strength for these parents and families. Pediatricians and other primary health care professionals who care for children are at the forefront of primary care delivery; they are the frontline providers that families encounter upon entry to the health care system. Given their pivotal position in the frontline of health care delivery, pediatricians, specifically, can foster educational and practice programs aimed at increasing partnership between families and their health care providers. Delivering health care that is centered on people's needs and expectations is a critical element in primary health care's mission of better health for all [19]. Providing care that is family-centered is a key element of this mission and may also be pivotal to reducing and ultimately eliminating health disparities among all populations regardless of geography, socioeconomic status, or racial/ethnic minority group.

## Figures and Tables

**Table 1:** Observed (unadjusted) prevalence of not receiving family-centered care according to US state and selected sociodemographic characteristics: the 2011-2012 National Survey of Children's Health (*N* = 91,001).

Covariate	* *No family-centered care		* *Not spending enough time		* *Does not listen carefully		* *Not sensitive to values		* *Not providing information		* *Not feeling like a partner	
	Weighted %	SE	Weighted %	SE	Weighted %	SE	Weighted %	SE	Weighted %	SE	Weighted %	SE
*United States*	33.41	0.37	22.51	0.34	10.62	0.25	10.84	0.27	14.74	0.29	12.27	0.27
*State of residence*												
Alaska	31.99	1.68	21.21	1.48	10.17	1.11	11.08	1.19	15.44	1.33	12.61	1.22
Alabama	34.27	1.69	22.89	1.55	10.87	1.18	10.51	1.09	13.58	1.22	11.81	1.16
Arkansas	32.76	1.73	22.60	1.59	10.80	1.17	9.36	1.09	12.62	1.19	10.79	1.16
Arizona	40.98	1.76	30.11	1.70	13.60	1.27	13.38	1.27	19.28	1.45	15.81	1.32
California	42.23	1.81	29.90	1.68	14.63	1.27	15.95	1.37	21.23	1.50	16.70	1.35
Colorado	31.27	1.75	18.94	1.52	11.38	1.23	10.76	1.19	15.25	1.39	11.32	1.21
Connecticut	29.15	1.41	18.67	1.23	8.56	0.89	7.39	0.80	11.30	0.98	9.08	0.86
District of Columbia	36.92	2.07	23.31	1.82	9.49	1.25	11.84	1.40	14.51	1.51	11.65	1.40
Delaware	32.59	1.69	21.12	1.50	8.14	1.02	8.11	1.04	12.10	1.20	11.28	1.17
Florida	37.84	1.74	26.92	1.63	12.40	1.24	11.83	1.17	17.00	1.35	15.14	1.33
Georgia	34.85	1.69	23.75	1.54	11.08	1.16	10.94	1.13	13.08	1.17	13.63	1.23
Hawaii	32.52	1.50	21.38	1.31	9.13	0.94	10.15	0.99	15.74	1.17	11.16	1.00
Iowa	22.44	1.38	14.15	1.17	6.61	0.83	6.13	0.84	10.18	0.99	7.70	0.89
Idaho	28.92	1.72	19.21	1.54	7.13	0.92	6.87	0.92	13.62	1.28	10.27	1.12
Illinois	32.32	1.56	22.23	1.42	8.88	0.96	10.46	1.07	14.23	1.16	10.89	1.02
Indiana	28.98	1.61	18.23	1.39	8.61	1.05	8.04	1.00	13.55	1.24	9.56	1.10
Kansas	30.25	1.57	19.94	1.40	8.75	0.96	7.99	0.96	13.04	1.16	9.68	0.98
Kentucky	29.00	1.53	18.15	1.33	9.19	0.99	8.79	0.98	11.97	1.06	10.54	1.03
Louisiana	33.22	1.71	23.30	1.59	9.02	1.04	8.73	1.03	14.60	1.30	12.30	1.20
Massachusetts	26.04	1.42	16.53	1.24	5.17	0.69	7.02	0.90	9.64	0.87	8.06	0.88
Maryland	31.79	1.67	21.65	1.52	10.70	1.12	9.11	1.04	12.71	1.17	11.24	1.15
Maine	25.18	1.42	14.93	1.23	8.16	0.94	5.77	0.77	10.26	0.92	9.29	0.96
Michigan	27.60	1.59	18.30	1.40	7.24	0.88	7.04	0.90	11.42	1.10	10.24	1.10
Minnesota	28.04	1.60	18.08	1.41	8.96	1.06	8.59	1.08	13.76	1.28	10.15	1.10
Missouri	26.03	1.46	16.38	1.23	8.49	0.93	6.18	0.78	10.34	0.98	8.81	0.95
Mississippi	39.64	1.85	29.04	1.74	14.38	1.36	15.16	1.39	17.22	1.47	15.94	1.42
Montana	30.09	1.58	18.19	1.34	10.68	1.08	8.89	1.02	14.10	1.19	11.37	1.07
North Carolina	32.60	1.76	23.88	1.63	8.33	1.04	7.04	0.95	10.99	1.13	9.23	1.09
North Dakota	26.32	1.60	14.12	1.24	7.58	0.96	6.55	0.93	11.65	1.13	7.88	0.98
Nebraska	27.64	1.47	18.20	1.32	7.33	0.85	7.45	0.92	11.66	1.04	8.62	0.96
New Hampshire	23.95	1.45	12.18	1.11	6.60	0.85	5.88	0.81	9.38	0.95	9.52	1.01
New Jersey	34.57	1.60	21.43	1.42	8.91	0.95	10.75	1.10	16.06	1.25	14.17	1.19
New Mexico	41.30	1.88	29.61	1.79	13.57	1.32	13.84	1.38	17.04	1.43	14.42	1.36
Nevada	41.82	1.87	29.77	1.74	16.57	1.45	16.54	1.44	21.32	1.55	17.33	1.43
New York	36.19	1.52	22.36	1.34	10.30	1.00	13.46	1.13	17.49	1.22	13.62	1.11
Ohio	28.64	1.62	18.01	1.39	8.55	1.04	7.88	1.01	11.73	1.17	10.90	1.20
Oklahoma	30.31	1.56	20.25	1.39	12.09	1.14	9.30	1.00	12.87	1.11	12.72	1.16
Oregon	31.46	1.60	19.57	1.42	11.19	1.15	9.52	1.09	12.81	1.13	10.92	1.10
Pennsylvania	28.88	1.75	17.60	1.49	8.38	1.09	7.00	0.98	10.99	1.17	9.23	1.12
Rhode Island	28.45	1.51	17.83	1.30	7.97	0.95	9.25	1.04	12.53	1.13	10.54	1.06
South Carolina	32.12	1.62	20.53	1.38	10.20	1.05	10.88	1.11	14.65	1.23	11.46	1.12
South Dakota	27.42	1.48	18.56	1.32	8.18	0.89	8.05	0.92	11.71	1.04	9.21	0.96
Tennessee	28.39	1.63	19.95	1.46	9.72	1.09	8.49	1.02	11.53	1.15	10.03	1.14
Texas	36.50	1.79	26.63	1.66	13.64	1.31	15.03	1.38	15.69	1.35	13.81	1.30
Utah	23.72	1.41	15.69	1.24	6.71	0.80	5.59	0.81	12.16	1.10	8.27	0.92
Virginia	33.31	1.73	22.09	1.58	10.76	1.18	10.06	1.17	13.35	1.24	11.27	1.19
Vermont	19.36	1.28	9.80	1.00	5.21	0.74	5.64	0.79	8.75	0.87	6.86	0.82
Washington	29.12	1.61	16.65	1.31	10.00	1.11	9.66	1.06	13.33	1.21	11.79	1.18
Wisconsin	24.72	1.45	15.84	1.27	6.45	0.81	6.91	0.87	10.15	1.01	6.42	0.77
West Virginia	26.06	1.39	14.89	1.14	9.23	0.97	8.75	0.94	9.90	0.93	9.21	0.93
Wyoming	27.53	1.47	15.03	1.16	9.03	0.95	6.75	0.83	12.09	1.05	8.37	0.90
*Child's age, y*												
0–5	30.76	0.64	21.09	0.59	8.92	0.39	9.81	0.43	13.39	0.47	10.37	0.42
6–11	34.64	0.65	23.46	0.59	11.20	0.46	11.19	0.46	15.83	0.52	13.01	0.48
12–17	34.72	0.64	22.94	0.58	11.68	0.45	11.46	0.48	15.90	0.49	13.37	0.47
*Child's sex*												
Male	33.63	0.51	22.92	0.48	10.82	0.36	11.37	0.38	15.02	0.41	12.77	0.39
Female	33.18	0.53	22.09	0.48	10.41	0.35	10.28	0.37	14.45	0.41	11.76	0.37
*Race/ethnicity*												
Hispanic	49.98	1.05	39.12	1.01	17.16	0.78	20.03	0.86	21.58	0.87	17.85	0.80
Non-Hispanic White	22.29	0.37	12.24	0.30	6.49	0.22	5.40	0.21	9.88	0.28	8.08	0.26
Non-Hispanic Black	43.76	1.03	31.44	0.95	13.74	0.75	14.25	0.72	18.07	0.82	16.10	0.77
Mixed race	30.10	1.41	18.30	1.24	11.00	0.99	9.51	0.90	13.17	1.02	10.58	0.87
Other	45.04	1.50	31.20	1.40	13.96	1.06	15.81	1.10	22.91	1.22	18.90	1.15
*Household composition*												
Two-parent biological household	30.33	0.44	19.91	0.40	9.05	0.29	9.47	0.31	13.54	0.34	10.76	0.31
Two-parent stepfamily	37.72	1.35	27.57	1.33	12.99	1.00	11.79	1.02	13.97	0.99	14.24	1.08
Single mother	40.42	0.88	28.19	0.82	13.60	0.65	14.34	0.68	18.57	0.72	15.21	0.67
Other family types	37.85	1.44	25.22	1.30	14.21	1.08	12.88	0.90	16.53	1.13	15.97	1.03
*Child health status*												
CSHCN	32.97	0.78	20.22	0.68	11.97	0.58	11.11	0.57	15.88	0.62	13.22	0.59
No CSHCN	33.52	0.42	23.11	0.39	10.28	0.28	10.77	0.30	14.45	0.32	12.03	0.30
*Place of residence*												
Metropolitan	45.83	0.43	22.68	0.39	10.53	0.29	10.94	0.30	14.98	0.33	12.44	0.30
Nonmetropolitan	44.52	0.74	21.85	0.66	11.07	0.53	10.35	0.55	13.63	0.55	11.49	0.55
*Primary language spoken at home*												
English	28.44	0.36	17.64	0.31	8.77	0.23	8.08	0.23	12.28	0.27	10.40	0.25
Any other language	62.03	1.24	50.51	1.26	21.23	1.03	26.63	1.13	28.71	1.14	22.94	1.05
*Household/parental education level, y*												
<12	58.65	1.43	46.59	1.46	21.88	1.23	25.87	1.32	26.55	1.30	21.48	1.22
12	42.63	0.94	31.38	0.90	13.70	0.67	14.26	0.71	17.03	0.74	14.71	0.68
>12	26.33	0.39	15.67	0.32	7.80	0.24	7.27	0.25	11.98	0.29	9.91	0.27
*Household poverty status [ratio of family income to poverty threshold]*												
Below 100%	50.60	0.89	39.15	0.90	18.40	0.73	19.82	0.76	22.30	0.78	19.21	0.74
100–199%	40.17	0.88	28.08	0.83	14.11	0.66	14.42	0.69	18.23	0.74	15.15	0.68
200–399%	28.02	0.67	17.42	0.60	7.62	0.39	7.62	0.42	12.10	0.48	9.76	0.44
At or above 400%	20.81	0.58	10.97	0.45	5.15	0.33	4.60	0.32	29.04	0.39	7.39	0.36
*Health insurance status at time of survey*												
Private insurance	25.10	0.42	52.60	2.00	33.68	2.07	29.53	2.05	35.31	2.01	30.12	1.95
Public insurance	43.14	0.68	31.19	0.64	13.73	0.48	15.28	0.52	18.03	0.53	15.31	0.50
No insurance	63.59	1.90	14.84	0.36	6.96	0.25	6.68	0.26	11.10	0.31	9.04	0.29

The chi-square test for the overall association between each covariate and the family-centered care outcomes was statistically significant at *p* < 0.05 level, except sex and child health status.

**Table 2:** Unadjusted odds of not receiving family-centered care by state, socioeconomic, and demographic characteristics: the 2011-2012 National Survey of Children's Health.

State	No family-centered care			Not spending enough time			Does not listen carefully			* *Not sensitive to values			Not providing information			Not feeling like partner		
	OR	95% CI		OR	95% CI		OR	95% CI		OR	95% CI		OR	95% CI		OR	95% CI	
Alaska	1.96	1.57	2.44	2.48	1.87	3.28	2.06	1.41	3.01	2.08	1.43	3.04	1.91	1.42	2.55	1.96	1.41	2.73
Alabama	2.17	1.75	2.70	2.73	2.06	3.62	2.22	1.52	3.24	1.97	1.36	2.85	1.64	1.22	2.20	1.82	1.30	2.54
Arkansas	2.03	1.62	2.53	2.69	2.02	3.57	2.20	1.51	3.22	1.73	1.17	2.54	1.51	1.12	2.04	1.64	1.16	2.32
Arizona	2.89	2.33	3.59	3.96	3.02	5.21	2.87	1.99	4.12	2.58	1.80	3.72	2.49	1.88	3.30	2.55	1.85	3.51
California	3.05	2.45	3.78	3.92	2.99	5.15	3.12	2.19	4.45	3.18	2.23	4.53	2.81	2.13	3.71	2.72	1.99	3.73
Colorado	1.90	1.51	2.38	2.15	1.60	2.89	2.34	1.60	3.42	2.02	1.38	2.95	1.88	1.39	2.54	1.73	1.23	2.45
Connecticut	1.71	1.39	2.11	2.11	1.61	2.78	1.71	1.18	2.47	1.34	0.92	1.94	1.33	1.00	1.77	1.36	0.98	1.88
District of Columbia	2.44	1.92	3.09	2.80	2.07	3.77	1.91	1.27	2.88	2.25	1.52	3.33	1.77	1.29	2.44	1.79	1.24	2.58
Delaware	2.01	1.62	2.51	2.46	1.85	3.27	1.61	1.08	2.40	1.48	0.99	2.20	1.44	1.06	1.95	1.73	1.23	2.43
Florida	2.54	2.04	3.15	3.39	2.57	4.46	2.58	1.78	3.73	2.25	1.56	3.24	2.14	1.61	2.84	2.42	1.75	3.35
Georgia	2.23	1.79	2.77	2.87	2.17	3.78	2.27	1.56	3.30	2.06	1.42	2.98	1.57	1.17	2.11	2.14	1.55	2.97
Hawaii	2.01	1.63	2.47	2.50	1.91	3.28	1.83	1.26	2.65	1.89	1.32	2.71	1.95	1.48	2.57	1.71	1.24	2.35
Iowa	1.21	0.96	1.51	1.52	1.13	2.03	1.29	0.87	1.91	1.09	0.73	1.64	1.18	0.87	1.60	1.13	0.80	1.61
Idaho	1.70	1.35	2.13	2.19	1.63	2.94	1.40	0.94	2.09	1.23	0.82	1.85	1.64	1.22	2.23	1.55	1.10	2.20
Illinois	1.99	1.61	2.46	2.63	2.00	3.46	1.77	1.22	2.58	1.95	1.35	2.82	1.73	1.30	2.30	1.66	1.20	2.30
Indiana	1.70	1.36	2.12	2.05	1.54	2.73	1.72	1.16	2.54	1.46	0.98	2.17	1.64	1.21	2.20	1.44	1.01	2.04
Kansas	1.81	1.45	2.24	2.29	1.73	3.04	1.75	1.20	2.55	1.45	0.99	2.14	1.56	1.17	2.10	1.46	1.04	2.03
Kentucky	1.70	1.37	2.11	2.04	1.54	2.71	1.84	1.27	2.68	1.61	1.10	2.35	1.42	1.06	1.90	1.60	1.15	2.23
Louisiana	2.07	1.66	2.58	2.79	2.11	3.71	1.81	1.23	2.66	1.60	1.09	2.36	1.78	1.33	2.40	1.90	1.37	2.66
Massachusetts	1.47	1.18	1.82	1.82	1.37	2.42	0.99	0.66	1.49	1.26	0.85	1.88	1.11	0.83	1.49	1.19	0.85	1.68
Maryland	1.94	1.56	2.42	2.54	1.91	3.37	2.18	1.50	3.17	1.68	1.14	2.46	1.52	1.13	2.04	1.72	1.23	2.41
Maine	1.40	1.13	1.74	1.61	1.21	2.16	1.62	1.10	2.37	1.02	0.68	1.53	1.19	0.89	1.59	1.39	0.99	1.95
Michigan	1.59	1.27	1.99	2.06	1.54	2.75	1.42	0.96	2.10	1.27	0.85	1.89	1.34	0.99	1.82	1.55	1.10	2.18
Minnesota	1.62	1.30	2.03	2.03	1.52	2.72	1.79	1.21	2.65	1.57	1.06	2.34	1.67	1.23	2.25	1.53	1.09	2.17
Missouri	1.47	1.18	1.82	1.80	1.36	2.39	1.69	1.16	2.46	1.10	0.74	1.63	1.20	0.89	1.62	1.31	0.93	1.85
Mississippi	2.74	2.19	3.41	3.77	2.85	4.97	3.06	2.12	4.41	2.99	2.08	4.29	2.17	1.62	2.91	2.58	1.86	3.57
Montana	1.79	1.44	2.23	2.05	1.54	2.72	2.18	1.51	3.15	1.63	1.11	2.39	1.71	1.28	2.28	1.74	1.26	2.42
North Carolina	2.01	1.61	2.52	2.89	2.17	3.83	1.65	1.11	2.46	1.27	0.84	1.91	1.29	0.94	1.76	1.38	0.97	1.97
North Dakota	1.49	1.19	1.87	1.51	1.12	2.04	1.49	1.00	2.22	1.17	0.77	1.78	1.38	1.02	1.86	1.16	0.81	1.67
Nebraska	1.59	1.28	1.97	2.05	1.54	2.71	1.44	0.98	2.11	1.35	0.91	2.00	1.38	1.03	1.84	1.28	0.91	1.81
New Hampshire	1.31	1.05	1.64	1.28	0.94	1.73	1.29	0.86	1.92	1.04	0.69	1.57	1.08	0.80	1.47	1.43	1.02	2.01
New Jersey	2.20	1.78	2.72	2.51	1.90	3.31	1.78	1.23	2.59	2.02	1.39	2.92	2.00	1.51	2.64	2.24	1.63	3.08
New Mexico	2.93	2.35	3.66	3.87	2.93	5.11	2.86	1.98	4.13	2.69	1.85	3.89	2.14	1.60	2.87	2.29	1.64	3.19
Nevada	2.99	2.40	3.73	3.90	2.96	5.14	3.62	2.52	5.18	3.32	2.32	4.74	2.83	2.13	3.74	2.85	2.07	3.92
New York	2.36	1.92	2.90	2.65	2.02	3.47	2.09	1.45	3.01	2.60	1.83	3.69	2.21	1.69	2.90	2.14	1.57	2.93
Ohio	1.67	1.34	2.09	2.02	1.51	2.70	1.70	1.15	2.53	1.43	0.96	2.14	1.39	1.02	1.89	1.66	1.17	2.36
Oklahoma	1.81	1.46	2.25	2.34	1.77	3.09	2.50	1.74	3.60	1.71	1.18	2.49	1.54	1.15	2.06	1.98	1.43	2.74
Oregon	1.91	1.54	2.37	2.24	1.68	2.97	2.29	1.58	3.33	1.76	1.20	2.58	1.53	1.15	2.05	1.66	1.19	2.33
Pennsylvania	1.69	1.34	2.13	1.97	1.46	2.65	1.66	1.11	2.49	1.26	0.83	1.91	1.29	0.94	1.77	1.38	0.96	1.99
Rhode Island	1.66	1.33	2.06	2.00	1.50	2.65	1.58	1.07	2.33	1.70	1.16	2.50	1.49	1.11	2.01	1.60	1.15	2.24
South Carolina	1.97	1.59	2.45	2.38	1.80	3.14	2.07	1.43	3.00	2.04	1.41	2.95	1.79	1.34	2.39	1.76	1.26	2.45
South Dakota	1.57	1.27	1.95	2.10	1.58	2.77	1.62	1.12	2.36	1.47	1.00	2.14	1.38	1.03	1.85	1.38	0.98	1.93
Tennessee	1.65	1.32	2.07	2.29	1.72	3.05	1.96	1.34	2.87	1.55	1.05	2.29	1.36	1.00	1.85	1.51	1.06	2.16
Texas	2.39	1.92	2.98	3.34	2.53	4.41	2.88	1.99	4.15	2.96	2.06	4.25	1.94	1.45	2.60	2.18	1.56	3.03
Utah	1.30	1.04	1.62	1.71	1.28	2.28	1.31	0.89	1.93	0.99	0.65	1.51	1.44	1.08	1.94	1.22	0.87	1.73
Virginia	2.08	1.67	2.60	2.61	1.96	3.47	2.20	1.50	3.21	1.87	1.27	2.75	1.61	1.19	2.17	1.73	1.22	2.43
Vermont	1.00		Reference	1.00		Reference	1.00		Reference	1.00		Reference	1.00		Reference	1.00		Reference
Washington	1.71	1.37	2.14	1.84	1.38	2.45	2.02	1.38	2.96	1.79	1.23	2.61	1.60	1.19	2.16	1.82	1.30	2.54
Wisconsin	1.37	1.10	1.71	1.73	1.30	2.32	1.26	0.84	1.87	1.24	0.84	1.84	1.18	0.87	1.60	0.93	0.65	1.33
West Virginia	1.47	1.19	1.82	1.61	1.21	2.14	1.85	1.28	2.69	1.60	1.11	2.33	1.15	0.85	1.54	1.38	0.99	1.92
Wyoming	1.58	1.28	1.96	1.63	1.22	2.16	1.81	1.25	2.62	1.21	0.82	1.79	1.43	1.07	1.92	1.24	0.88	1.74
*Child's age, y*																		
0–5	1.00		Reference	1.00		Reference	1.00		Reference	1.00		Reference	1.00		Reference	1.00		Reference
6–11	1.19	1.10	1.29	1.15	1.04	1.26	1.29	1.13	1.47	1.16	1.02	1.32	1.33	1.19	1.49	1.29	1.14	1.46
12–17	1.20	1.10	1.30	1.11	1.01	1.22	1.35	1.19	1.54	1.19	1.04	1.36	1.34	1.20	1.50	1.33	1.18	1.50
*Child's sex*																		
Male	1.02	0.96	1.09	1.05	0.97	1.13	1.04	0.94	1.16	1.12	1.00	1.25	1.05	0.96	1.15	1.10	1.00	1.21
Female	1.00		Reference	1.00		Reference	1.00		Reference	1.00		Reference	1.00		Reference	1.00		Reference
*Race/ethnicity*																		
Non-Hispanic White	1.00		Reference	1.00		Reference	1.00		Reference	1.00		Reference	1.00		Reference	1.00		Reference
Non-Hispanic Black	2.71	2.47	2.97	3.29	2.97	3.64	2.30	1.99	2.65	2.91	2.53	3.35	2.01	1.78	2.28	2.18	1.91	2.49
Hispanic	3.48	3.18	3.82	4.60	4.17	5.09	2.99	2.62	3.40	4.39	3.84	5.02	2.51	2.23	2.82	2.47	2.18	2.81
Non-Hispanic mixed race	1.50	1.31	1.72	1.61	1.35	1.91	1.78	1.44	2.20	1.84	1.48	2.29	1.38	1.15	1.66	1.35	1.11	1.63
Other	2.86	2.52	3.24	3.25	2.83	3.74	2.34	1.94	2.82	3.29	2.75	3.94	2.71	2.34	3.14	2.65	2.26	3.11
*Household composition*																		
Two-parent biological household	1.00		Reference	1.00		Reference	1.00		Reference	1.00		Reference	1.00		Reference	1.00		Reference
Two-parent stepfamily	1.39	1.23	1.57	1.53	1.33	1.76	1.50	1.25	1.81	1.28	1.04	1.57	1.04	0.87	1.23	1.38	1.15	1.66
Single mother	1.56	1.43	1.69	1.58	1.44	1.73	1.58	1.39	1.80	1.60	1.40	1.82	1.46	1.31	1.63	1.49	1.32	1.68
Other family type	1.40	1.23	1.59	1.36	1.17	1.57	1.66	1.38	2.01	1.41	1.19	1.68	1.27	1.07	1.50	1.58	1.34	1.86
*Place of residence*																		
Metropolitan	1.00		Reference	1.00		Reference	1.00		Reference	1.00		Reference	1.00		Reference	1.00		Reference
Nonmetropolitan	0.97	0.90	1.04	0.96	0.88	1.04	1.06	0.94	1.19	0.94	0.82	1.07	0.90	0.81	0.99	0.91	0.81	1.03
*Primary language spoken at home*																		
English	1.00		Reference	1.00		Reference	1.00		Reference	1.00		Reference	1.00		Reference	1.00		Reference
Any other language	4.11	3.69	4.58	4.76	4.28	5.30	2.81	2.45	3.21	4.13	3.63	4.70	2.88	2.55	3.24	2.57	2.26	2.91
*Household/parental education level, y*																		
<12	3.97	3.51	4.49	4.69	4.15	5.32	3.31	2.84	3.87	4.45	3.82	5.18	2.66	2.31	3.06	2.49	2.13	2.90
12	2.08	1.91	2.26	2.46	2.24	2.71	1.88	1.65	2.13	2.12	1.85	2.42	1.51	1.34	1.69	1.57	1.39	1.77
12+	1.00		Reference	1.00		Reference	1.00		Reference	1.00		Reference	1.00		Reference	1.00		Reference
*Household poverty status (ratio of family income to poverty threshold)*																		
Below 100%	3.90	3.53	4.31	5.22	4.66	5.86	4.15	3.52	4.9	5.13	4.33	6.09	2.89	2.54	3.28	2.98	2.60	3.42
100–199%	2.56	2.31	2.83	3.17	2.80	3.59	3.03	2.53	3.61	3.50	2.90	4.22	2.24	1.95	2.58	2.24	1.93	2.60
200–399%	1.48	1.34	1.63	1.71	1.51	1.95	1.52	1.28	1.80	1.71	1.42	2.06	1.39	1.22	1.58	1.36	1.18	1.56
At or above 400%	1.00		Reference	1.00		Reference	1.00		Reference	1.00		Reference	1.00		Reference	1.00		Reference
*Health insurance status at time of survey*																		
Private insurance	1.00		Reference	1.00		Reference	1.00		Reference	1.00		Reference	1.00		Reference	1.00		Reference
Public insurance	2.26	2.11	2.43	2.60	2.40	2.82	2.13	1.91	2.37	2.52	2.25	2.82	1.76	1.60	1.94	1.82	1.64	2.02
No insurance	5.21	4.41	6.16	6.37	5.39	7.53	6.79	5.58	8.27	5.85	4.74	7.22	4.37	3.64	5.25	4.34	3.57	5.27

**Table 3:** State variations in selected socioeconomic and demographic characteristics (weighted percentages), United States: the 2011-2012 National Survey of Children's Health.

State	Non-White minority children	Single mother households	Non-English-speaking households	Household or parental education < 12 years	Households below poverty level	Children without health insurance
*United States*	48.86	18.85	15.61	11.44	22.46	5.49
Alaska	46.83	16.48	5.43	5.29	19.88	5.79
Alabama	44.80	24.99	4.43	10.86	26.87	4.01
Arkansas	40.03	20.67	8.37	10.52	26.74	4.64
Arizona	59.74	18.82	21.99	14.71	28.89	11.70
California	73.19	14.17	35.35	18.66	24.27	6.32
Colorado	43.07	16.60	15.39	9.68	16.77	7.59
Connecticut	42.99	17.77	15.42	8.10	14.56	2.64
District of Columbia	82.11	37.91	13.25	12.57	31.03	1.27
Delaware	50.85	21.95	9.76	9.42	18.39	3.65
Florida	57.36	22.43	15.94	10.13	26.26	9.32
Georgia	55.53	25.67	9.80	11.91	25.85	7.16
Hawaii	79.69	13.08	8.02	4.61	19.06	1.22
Iowa	20.75	15.88	6.25	6.31	17.54	2.74
Idaho	24.82	12.33	7.69	7.91	20.99	5.75
Illinois	48.79	18.98	19.58	10.74	20.90	1.57
Indiana	28.64	19.96	6.04	10.18	21.34	5.33
Kansas	34.33	14.45	10.98	9.83	19.19	5.01
Kentucky	24.51	21.40	3.25	9.01	28.58	4.18
Louisiana	51.55	28.70	3.34	11.77	28.21	2.08
Massachusetts	36.42	19.72	12.79	7.17	14.63	1.04
Maryland	55.98	17.99	11.53	7.83	14.40	4.36
Maine	12.00	14.53	1.39	3.89	16.71	3.80
Michigan	34.15	22.09	5.21	7.75	23.54	2.67
Minnesota	29.00	14.62	7.53	6.56	15.02	4.44
Missouri	29.74	19.88	4.01	7.83	21.32	4.26
Mississippi	54.14	30.29	3.18	10.58	32.75	7.27
Montana	23.39	15.21	1.48	4.05	18.19	8.55
North Carolina	47.27	21.62	10.47	10.80	25.67	6.16
North Dakota	24.58	13.49	1.20	2.21	11.81	6.50
Nebraska	30.03	15.69	10.40	8.00	16.33	4.98
New Hampshire	13.93	14.59	3.22	2.11	10.31	3.37
New Jersey	52.44	17.84	19.32	7.00	14.64	3.46
New Mexico	75.76	17.94	20.96	15.85	30.56	6.68
Nevada	63.41	19.15	27.10	18.86	24.04	13.25
New York	51.31	19.47	19.70	11.36	22.66	2.81
Ohio	27.74	20.33	3.55	8.15	23.40	3.20
Oklahoma	45.07	18.28	6.71	10.30	23.65	7.27
Oregon	37.22	14.58	14.62	11.68	23.96	4.32
Pennsylvania	32.13	17.89	7.53	8.64	20.29	4.15
Rhode Island	39.23	20.30	15.46	11.56	18.73	3.89
South Carolina	48.37	25.62	6.28	9.90	27.28	6.40
South Dakota	24.59	12.61	1.62	5.89	14.24	3.24
Tennessee	37.87	22.53	6.11	8.74	26.16	5.36
Texas	66.97	18.19	25.83	18.15	25.30	9.40
Utah	25.71	9.76	9.92	6.50	15.57	8.71
Virginia	46.29	15.97	12.43	6.94	15.53	5.27
Vermont	13.05	16.79	0.82	2.97	13.50	1.32
Washington	41.62	16.34	15.22	9.88	18.19	3.64
Wisconsin	29.56	18.79	6.63	8.00	17.00	1.65
West Virginia	13.07	19.31	0.54	8.81	25.16	4.17
Wyoming	22.67	13.84	4.22	3.72	10.08	5.90

**Table 4:** Adjusted^1^ odds of not receiving family-centered care by state, socioeconomic, and demographic characteristics: the 2011-2012 National Survey of Children's Health.

Covariate	No family-centered care			Not spending enough time			Does not listen carefully			Not sensitive to values			Not providing information			Not feeling like partner		
	AOR	95% CI		AOR	95% CI		AOR	95% CI		AOR	95% CI		AOR	95% CI		AOR	95% CI	
Alaska	1.36	1.08	1.72	1.55	1.16	2.09	1.36	0.92	2.02	1.27	0.86	1.89	1.34	0.99	1.82	1.36	0.97	1.93
Alabama	1.47	1.17	1.86	1.61	1.20	2.18	1.56	1.05	2.31	1.19	0.80	1.78	1.14	0.83	1.56	1.23	0.87	1.76
Arkansas	1.33	1.05	1.69	1.53	1.13	2.07	1.49	1.00	2.23	1.00	0.67	1.51	1.02	0.75	1.40	1.10	0.76	1.58
Arizona	1.65	1.29	2.11	1.81	1.33	2.47	1.63	1.09	2.45	1.20	0.80	1.80	1.45	1.05	1.99	1.47	1.03	2.10
California	1.56	1.21	2.00	1.57	1.15	2.15	1.90	1.28	2.82	1.40	0.92	2.12	1.59	1.15	2.19	1.57	1.09	2.25
Colorado	1.40	1.09	1.80	1.28	0.92	1.77	1.81	1.20	2.73	1.27	0.83	1.94	1.35	0.97	1.87	1.26	0.86	1.83
Connecticut	1.29	1.02	1.63	1.34	0.99	1.83	1.44	0.96	2.15	0.89	0.59	1.36	0.98	0.71	1.35	1.02	0.71	1.46
District of Columbia	1.19	0.91	1.57	1.09	0.78	1.53	1.15	0.73	1.80	1.02	0.65	1.58	1.00	0.69	1.43	0.99	0.66	1.49
Delaware	1.42	1.11	1.83	1.48	1.08	2.04	1.25	0.81	1.93	0.94	0.60	1.46	1.02	0.72	1.43	1.23	0.84	1.80
Florida	1.53	1.20	1.96	1.69	1.24	2.31	1.63	1.09	2.43	1.17	0.77	1.77	1.32	0.96	1.80	1.50	1.05	2.15
Georgia	1.35	1.06	1.72	1.45	1.06	1.97	1.44	0.96	2.15	1.09	0.72	1.65	0.96	0.69	1.33	1.33	0.93	1.90
Hawaii	1.29	1.01	1.64	1.42	1.04	1.94	1.29	0.85	1.96	1.08	0.72	1.64	1.20	0.87	1.65	1.06	0.73	1.52
Iowa	1.05	0.83	1.33	1.22	0.90	1.66	1.15	0.76	1.74	0.89	0.58	1.37	1.01	0.74	1.39	0.98	0.68	1.41
Idaho	1.34	1.05	1.71	1.56	1.14	2.14	1.06	0.70	1.59	0.87	0.57	1.33	1.36	1.00	1.86	1.28	0.90	1.82
Illinois	1.28	1.01	1.62	1.41	1.04	1.92	1.31	0.88	1.97	1.13	0.75	1.69	1.17	0.86	1.59	1.14	0.79	1.63
Indiana	1.31	1.02	1.67	1.39	1.02	1.91	1.34	0.89	2.04	1.03	0.67	1.58	1.25	0.90	1.72	1.09	0.74	1.59
Kansas	1.37	1.08	1.73	1.48	1.09	2.01	1.32	0.88	1.97	0.95	0.62	1.44	1.16	0.85	1.59	1.08	0.75	1.55
Kentucky	1.34	1.07	1.70	1.46	1.08	1.99	1.42	0.95	2.11	1.18	0.79	1.76	1.11	0.82	1.51	1.23	0.87	1.75
Louisiana	1.32	1.04	1.68	1.55	1.14	2.11	1.25	0.83	1.89	0.93	0.62	1.41	1.22	0.88	1.68	1.28	0.90	1.84
Massachusetts	1.19	0.94	1.52	1.31	0.95	1.79	0.89	0.58	1.38	0.94	0.61	1.46	0.87	0.63	1.20	0.95	0.65	1.38
Maryland	1.36	1.05	1.75	1.52	1.10	2.10	1.74	1.15	2.64	1.06	0.69	1.64	1.06	0.76	1.48	1.21	0.83	1.77
Maine	1.29	1.03	1.61	1.45	1.07	1.96	1.45	0.98	2.13	0.91	0.61	1.37	1.10	0.82	1.47	1.28	0.91	1.80
Michigan	1.20	0.94	1.52	1.40	1.02	1.90	1.13	0.75	1.71	0.88	0.58	1.35	1.01	0.73	1.39	1.16	0.80	1.66
Minnesota	1.37	1.08	1.73	1.54	1.14	2.10	1.56	1.04	2.35	1.21	0.79	1.86	1.33	0.97	1.83	1.24	0.86	1.78
Missouri	1.16	0.92	1.48	1.28	0.94	1.75	1.38	0.92	2.06	0.80	0.53	1.22	0.92	0.67	1.28	1.01	0.70	1.46
Mississippi	1.61	1.27	2.03	1.91	1.42	2.58	1.82	1.24	2.67	1.58	1.08	2.31	1.36	1.00	1.86	1.59	1.13	2.23
Montana	1.45	1.16	1.82	1.55	1.15	2.08	1.60	1.09	2.33	1.19	0.80	1.77	1.36	1.01	1.82	1.36	0.97	1.90
North Carolina	1.31	1.03	1.68	1.63	1.20	2.22	1.10	0.72	1.68	0.71	0.46	1.10	0.86	0.61	1.19	0.91	0.63	1.33
North Dakota	1.33	1.05	1.69	1.25	0.91	1.72	1.23	0.82	1.85	0.96	0.62	1.47	1.17	0.86	1.60	0.98	0.68	1.43
Nebraska	1.26	1.00	1.59	1.43	1.05	1.94	1.15	0.76	1.72	0.94	0.62	1.43	1.07	0.79	1.45	1.01	0.70	1.45
New Hampshire	1.35	1.06	1.73	1.24	0.90	1.72	1.39	0.90	2.14	1.06	0.68	1.67	0.99	0.71	1.39	1.35	0.92	1.96
New Jersey	1.56	1.22	1.98	1.49	1.09	2.03	1.44	0.96	2.17	1.30	0.86	1.96	1.42	1.04	1.95	1.64	1.15	2.34
New Mexico	1.49	1.17	1.91	1.57	1.15	2.14	1.53	1.03	2.28	1.13	0.76	1.70	1.23	0.89	1.69	1.27	0.88	1.83
Nevada	1.61	1.25	2.07	1.64	1.19	2.26	2.11	1.41	3.15	1.49	0.99	2.26	1.59	1.15	2.20	1.62	1.13	2.33
New York	1.51	1.20	1.91	1.38	1.02	1.87	1.50	1.01	2.23	1.49	1.01	2.21	1.45	1.07	1.97	1.43	1.01	2.03
Ohio	1.35	1.05	1.72	1.47	1.07	2.00	1.39	0.91	2.13	1.07	0.69	1.66	1.09	0.78	1.51	1.29	0.89	1.88
Oklahoma	1.23	0.97	1.56	1.39	1.02	1.88	1.71	1.17	2.51	1.03	0.69	1.53	1.06	0.78	1.45	1.36	0.96	1.92
Oregon	1.38	1.09	1.75	1.36	1.00	1.86	1.73	1.16	2.56	1.10	0.72	1.68	1.09	0.79	1.50	1.20	0.83	1.72
Pennsylvania	1.34	1.04	1.73	1.35	0.97	1.88	1.37	0.89	2.11	0.90	0.57	1.40	0.99	0.70	1.39	1.07	0.73	1.57
Rhode Island	1.19	0.93	1.52	1.20	0.87	1.65	1.24	0.81	1.88	1.08	0.71	1.66	1.06	0.76	1.47	1.16	0.80	1.68
South Carolina	1.22	0.96	1.55	1.24	0.91	1.68	1.34	0.90	1.99	1.15	0.77	1.72	1.16	0.85	1.58	1.12	0.79	1.61
South Dakota	1.36	1.09	1.70	1.73	1.29	2.32	1.34	0.91	1.97	1.15	0.78	1.70	1.16	0.86	1.57	1.14	0.81	1.62
Tennessee	1.12	0.88	1.43	1.36	1.00	1.85	1.34	0.89	2.02	0.95	0.62	1.45	0.93	0.67	1.30	1.03	0.70	1.51
Texas	1.23	0.95	1.58	1.35	0.99	1.84	1.66	1.11	2.48	1.33	0.89	1.99	1.11	0.80	1.53	1.26	0.87	1.81
Utah	1.06	0.83	1.35	1.22	0.89	1.68	1.06	0.69	1.62	0.72	0.45	1.13	1.11	0.81	1.53	0.95	0.66	1.39
Virginia	1.56	1.22	1.99	1.70	1.24	2.33	1.76	1.17	2.65	1.26	0.83	1.91	1.17	0.84	1.61	1.27	0.88	1.84
Vermont	1.00		Reference	1.00		Reference	1.00		Reference	1.00		Reference	1.00		Reference	1.00		Reference
Washington	1.22	0.95	1.57	1.07	0.78	1.48	1.57	1.04	2.37	1.13	0.75	1.72	1.14	0.82	1.58	1.32	0.91	1.91
Wisconsin	1.12	0.88	1.43	1.28	0.93	1.76	1.09	0.72	1.66	0.94	0.61	1.44	0.95	0.69	1.32	0.76	0.52	1.11
West Virginia	1.28	1.02	1.60	1.33	0.99	1.79	1.61	1.09	2.36	1.37	0.93	2.03	0.98	0.72	1.34	1.18	0.83	1.67
Wyoming	1.40	1.12	1.76	1.32	0.98	1.78	1.54	1.05	2.25	0.98	0.66	1.47	1.29	0.96	1.73	1.11	0.78	1.57
*Child's age, y*																		
0–5	1.00		Reference	1.00		Reference	1.00		Reference	1.00		Reference	1.00		Reference	1.00		Reference
6–11	1.24	1.13	1.35	1.18	1.07	1.31	1.28	1.12	1.47	1.18	1.02	1.35	1.37	1.22	1.55	1.29	1.14	1.47
12–17	1.36	1.24	1.49	1.27	1.15	1.41	1.44	1.25	1.65	1.33	1.16	1.53	1.48	1.32	1.66	1.41	1.25	1.60
*Child's sex*																		
Male	1.01	0.95	1.09	1.04	0.96	1.13	1.02	0.92	1.13	1.11	0.99	1.24	1.04	0.95	1.14	1.09	0.99	1.20
Female	1.00		Reference	1.00		Reference	1.00		Reference	1.00		Reference	1.00		Reference	1.00		Reference
*Race/ethnicity*																		
Non-Hispanic White	1.00		Reference	1.00		Reference	1.00		Reference	1.00		Reference	1.00		Reference	1.00		Reference
Non-Hispanic Black	2.11	1.90	2.34	2.48	2.21	2.79	1.68	1.43	1.98	2.11	1.79	2.49	1.59	1.38	1.83	1.65	1.42	1.93
Hispanic	1.58	1.39	1.79	1.91	1.66	2.19	1.42	1.18	1.73	1.69	1.39	2.07	1.18	0.99	1.40	1.27	1.05	1.53
Non-Hispanic mixed race	1.38	1.19	1.59	1.45	1.21	1.74	1.57	1.28	1.94	1.62	1.31	2.01	1.26	1.05	1.52	1.21	0.99	1.46
Other	2.23	1.92	2.59	2.38	2.01	2.83	1.88	1.52	2.33	2.40	1.92	3.00	2.10	1.76	2.50	2.16	1.79	2.61
*Household composition*																		
Two-parent biological household	1.00		Reference	1.00		Reference	1.00		Reference	1.00		Reference	1.00		Reference	1.00		Reference
Two-parent stepfamily	1.28	1.13	1.45	1.48	1.27	1.71	1.33	1.10	1.60	1.22	0.98	1.51	0.97	0.81	1.15	1.27	1.05	1.55
Single mother	1.05	0.95	1.15	1.02	0.91	1.14	1.10	0.95	1.27	1.11	0.95	1.29	1.14	1.00	1.29	1.12	0.97	1.29
Other family type	1.12	0.97	1.29	1.08	0.91	1.28	1.37	1.12	1.68	1.20	0.99	1.45	1.11	0.92	1.33	1.37	1.15	1.64
*Place of residence*																		
Metropolitan	1.00		Reference	1.00		Reference	1.00		Reference	1.00		Reference	1.00		Reference	1.00		Reference
Nonmetropolitan	1.03	0.93	1.13	1.01	0.90	1.12	1.15	0.99	1.34	1.10	0.92	1.31	0.95	0.83	1.08	0.95	0.82	1.10
*Primary language spoken at home*																		
English	1.00		Reference	1.00		Reference	1.00		Reference	1.00		Reference	1.00		Reference	1.00		Reference
Any other language	2.23	1.93	2.58	2.35	2.02	2.73	1.49	1.23	1.82	1.97	1.61	2.42	1.85	1.55	2.20	1.64	1.36	1.98
*Household/parental education level, y*																		
<12	1.54	1.33	1.79	1.60	1.37	1.86	1.26	1.04	1.53	1.45	1.19	1.75	1.19	1.00	1.41	1.10	0.91	1.33
12	1.31	1.19	1.44	1.44	1.29	1.61	1.08	0.94	1.25	1.17	1.00	1.37	1.00	0.88	1.14	1.01	0.88	1.16
12+	1.00		Reference	1.00		Reference	1.00		Reference	1.00		Reference	1.00		Reference	1.00		Reference
*Household poverty status (ratio of family income to poverty threshold)*																		
Below 100%	1.96	1.69	2.26	2.29	1.95	2.69	2.58	2.03	3.28	2.49	1.94	3.18	1.98	1.66	2.37	2.08	1.72	2.52
100–199%	1.68	1.48	1.91	1.86	1.60	2.16	2.17	1.74	2.70	2.22	1.75	2.81	1.79	1.51	2.12	1.78	1.48	2.13
200–399%	1.34	1.21	1.49	1.48	1.30	1.69	1.38	1.15	1.65	1.55	1.28	1.88	1.35	1.19	1.53	1.31	1.13	1.52
At or above 400%	1.00		Reference	1.00		Reference	1.00		Reference	1.00		Reference	1.00		Reference	1.00		Reference
*Health insurance status at time of survey*																		
Private insurance	1.00		Reference	1.00		Reference	1.00		Reference	1.00		Reference	1.00		Reference	1.00		Reference
Public insurance	1.06	0.96	1.18	1.03	0.92	1.16	0.99	0.85	1.16	1.04	0.89	1.22	1.00	0.88	1.14	1.01	0.88	1.16
No insurance	2.83	2.37	3.36	3.03	2.53	3.61	3.49	2.81	4.33	2.63	2.09	3.32	2.62	2.15	3.19	2.55	2.06	3.14

^1^Adjusted by multivariate logistic regression for state, child's age, sex, race/ethnicity, household language use, household composition, metropolitan/nonmetropolitan residence, parental education, household poverty status, and health insurance status.

## References

[1] Kuo D. Z., Houtrow A. J., Arango P., Kuhlthau K. A., Simmons J. M., Neff J. M. (2012). Family-centered care: current applications and future directions in pediatric health care. *Maternal and Child Health Journal*.

[2] Institute for Patient-and Family-Centered Care Frequently Asked Questions. http://www.ipfcc.org/faq.html.

[3] Strickland B. B., Jones J. R., Ghandour R. M., Kogan M. D., Newacheck P. W. (2011). The medical home: health care access and impact for children and youth in the United States. *Pediatrics*.

[4] US Department of Health and Human Services (2014). *The Health and Well-Being of Children: A Portrait of States and the Nation, 2011-2012*.

[5] Committee on Hospital Care (2012). Patient- and family-centered care and the pediatrician's role (policy statement). *Pediatrics*.

[6] Institute of Medicine (IOM) (2001). *Crossing the Quality Chasm*.

[7] US Department of Health and Human Services Healthy People 2020. http://www.healthypeople.gov/2020/topics-objectives/topic/Access-to-Health-Services.

[8] Singh G. K., Strickland B. B., Ghandour R. M., van Dyck P. C. (2009). Geographic disparities in access to the medical home among US CSHCN. *Pediatrics*.

[9] Johnson B. H. (2000). Family-centered care: four decades of progress. *Families, Systems and Health*.

[10] Macinko J., Starfield B., Shi L. (2003). The contribution of primary care systems to health outcomes within Organization for Economic Cooperation and Development (OECD) countries, 1970–1998. *Health Services Research*.

[11] Zajicek-Farber M. L., Lotrecchiano G. R., Long T. M., Farber J. M. (2015). Parental perceptions of family centered care in medical homes of children with neurodevelopmental disabilities. *Maternal and Child Health Journal*.

[12] Kuo D. Z., Bird T. M., Tilford J. M. (2011). Associations of family-centered care with health care outcomes for children with special health care needs. *Maternal and Child Health Journal*.

[13] Hughes D. (2014). *A Review of the Literature Pertaining to Family-Centered Care for Children with Special Health Care Needs*.

[14] Antonelli R. C., McAllister J. W., Popp J. (2009). *Making Care Coordination a Critical Component of the Pediatirc Health Care System: A Multidisciplinary Framework*.

[15] U.S. Department of Health and Human Services (2015). *The Health and Well-Being of Children in Rural Areas: A Potrait of the Nation, 2011-2012*.

[16] Centers for Disease Control and Prevention 2011-2012 National Survey of Children's Health Frequently Asked Questions. http://www.cdc.gov/nchs/slaits/nsch.htm.

[17] Services USDoHaH 2014 Poverty Guidelines. http://aspe.hhs.gov/poverty/14poverty.cfm.

[18] SUDAAN (2009). *Software for the Statistical Analysis of Correlated Data, Release 10.01*.

[19] World Health Organization (2008). *The World Health Report 2008—Primary Health Care Now More Than Ever*.

[20] Organisation for Economic Cooperation and Development (2015). *Health Care Quality Indicators—Primary Care*.

[21] Health Council of the Netherlands (2004). European primary care. *Report no.: Publication*.

[22] Sambo L. G. (2011). Health systems and primary health care in the African region. *The African Health Monitor*.

[23] Agency for Healthcare Research and Quality (2014). Highlights: 2013 national healthcare quality & disparities reports. *Report*.

[24] Data Resource Center for Child & Adolescent Health Child Health Measures, Summary of Key Differences in Indicator Measurement: 2003 to 2007. http://www.childhealthdata.org/docs/nsch-docs/childhealthmeasures_03_vs_07_v2_508-pdf.pdf?sfvrsn=1.

[25] National Improvement Partnership Network (NIPN) https://www.uvm.edu/medicine/nipn/.

[26] The Henry J. Kaiser Family Foundation (2012). *Focus on Health Care Disparities: Key Facts*.

[27] Singh G. K., Schenker M. B., Xochitl C., Rodriguez-Lainz A. (2014). Use of national data systems to study immigrant health in the United States. *Migration and Health: A Research Methods Handbook*.

[28] Loue S., Wilson-Delfosse A., Limbach K. (2015). Identifying gaps in the cultural competence/sensitivity components of an undergraduate medical school curriculum: a needs assessment. *Journal of Immigrant and Minority Health*.

[29] Ryan C. (2013). Language use in the United States: 2011, American community survey reports.

[30] Family Voices (2008). *Family-Centered Care Self-Assessment Tool 2008*.

[31] Institute of Medicine (IOM) (2013). *Best Care at Lower Cost: The Path to Continuously Learning Health Care in America*.

[32] Shi L. (2012). The impact of primary care: a focused review. *Scientifica*.

